# Exploring new health markets: experiences from informal providers of transport for maternal health services in Eastern Uganda

**DOI:** 10.1186/1472-698X-11-S1-S10

**Published:** 2011-03-09

**Authors:** George W Pariyo, Chrispus Mayora, Olico Okui, Freddie Ssengooba, David H Peters, David Serwadda, Henry Lucas, Gerald Bloom, M Hafizur Rahman, Elizabeth Ekirapa-Kiracho

**Affiliations:** 1 Department of Health Policy, Planning and Management, Makerere University School of Public Health, P.O. Box 7072, Kampala, Uganda; 2 Department of International Health, Johns Hopkins Bloomberg School of Public Health, 615 North Wolfe Street, Baltimore, MD 21205, USA; 3 Department of Disease Control and Environmental Health, Makerere University School of Public Health, P.O. Box 7072, Kampala, Uganda; 4 Institute of Development Studies, at the University of Sussex, Brighton, BN1 9RE, UK

## Abstract

**Background:**

Although a number of intermediate transport initiatives have been used in some developing countries, available evidence reveals a dearth of local knowledge on the effect of these rural informal transport mechanisms on access to maternal health care services, the cost of implementing such schemes and their scalability. This paper, attempts to provide insights into the functioning of the informal transport markets in facilitating access to maternal health care. It also demonstrates the role that higher institutions of learning can play in designing projects that can increase the utilization of maternal health services.

**Objectives:**

To explore the use of intermediate transport mechanisms to improve access to maternal health services, with emphasis on the benefits and unintended consequences of the transport scheme, as well as challenges in the implementation of the scheme.

**Methods:**

This paper is based on the pilot phase to inform a quasi experimental study aimed at increasing access to maternal health services using demand and supply side incentives. The data collection for this paper included qualitative and quantitative methods that included focus group interviews, review of project documents and facility level data.

**Results:**

There was a marked increase in attendance of antenatal, and delivery care services, with the contracted transporters playing a leading role in mobilizing mothers to attend services. The project also had economic spill-over effects to the transport providers, their families and community generally. However, some challenges were faced including difficulty in setting prices for paying transporters, and poor enforcement of existing traffic regulations.

**Conclusions and implications:**

The findings indicate that locally existing resources such as motorcycle riders, also known as “boda boda” can be used innovatively to reduce challenges caused by geographical inaccessibility and a poor transport network with resultant increases in the utilization of maternal health services. However, care must be taken to mobilize the resources needed and to ensure that there is enforcement of laws that will ensure the safety of clients and the transport providers themselves.

## Background

The World Health Organization estimates that 75% of maternal deaths can be prevented through timely access to quality child-birth related care [1]. The ‘Three delays Model’ indicates that many maternal deaths occur at the first referral level either because of delays in making decisions to seek care, delays in reaching the facilities or delays in getting quality care at the facility [2,3]. Many studies in Uganda and other developing countries have indicated that the lack of access to transport as well as the cost of transport, both present challenges to accessing quality care at health facilities by women in labour and results in many mothers delivering at home by traditional birth attendants (TBAs) [4,5].

Several studies done in developing countries have concluded that innovative measures for facilitating geographical accessibility by providing alternative transport for women needing obstetric care amidst scarce healthcare resources could increase the level of maternal care service utilization and achieve equity in health [6-8]. For example, in Malawi, Uganda and Nigeria, motorcycle and bicycle ambulances have been used to provide emergency maternal transport services [7,8]. In Kebbi, Nigeria, a local bus Drivers’ union has been organized to provide transportation for women with obstetrical emergencies who later receive reimbursement for fuel costs through a fund created by the community [8]. Furthermore, these intermediate forms of transport have been reported to be relatively cheap when compared to the motorized ambulances [6-8].

A number of intermediate transport initiatives have been used in some developing countries although available evidence reveals a dearth of local knowledge on the impact of these rural informal transport mechanisms on access to maternal health care services, the cost of implementing such mechanisms and their scalability. A team from the Makerere University School of Public Health (MakSPH) under the Future Health Systems Research Program Consortium (FHS-RPC), and the Makerere University Johns Hopkins twinning program (MU-JHU), set out to implement a quasi experimental study using demand and supply side incentives to increase access to maternal health services in two districts in Eastern Uganda. This paper, attempts to provide insights from the pilot phase of this study into the functioning of the informal markets for transport in facilitating access to maternal health care. Specifically it explores the benefits and unintended consequences of the scheme, as well as challenges in implementation highlighting the implications for scaling up of such schemes. The findings from this exploratory research paper could guide implementers and funders who are interested in improving use of the informal transport market for maternal health services.

### Organization of transport services in Uganda

According to a survey done in Uganda, the most common locally available means of transport in rural areas were motor cycles and bicycles. Motorised vehicles such as buses, trucks and taxi’s are also used. However, they are more available in urban areas [6]. Transport for health care services including maternal health is through:

(a) Self provision through collective action independent of external agencies (citizens’ liaison systems). People often get together on a local basis as a community to transport patients, using, for example “make-shift stretchers” to transport mothers requiring emergency obstetric care to health facilities.

(b) Direct social provision, normally through state agencies. In some districts, there are government ambulances, at higher level health centres and district hospitals which provide transport, often for a subsidized fee upon notification especially for emergency cases. Often, however, patients are asked to provide fuel, usually in form of cash. Consequently only a few clients who can afford the required fuel contributions benefit since the majority of rural people are economically constrained.

(c) Direct-market provision. In this case, individuals purchase transport services from private providers. The providers include operators of bicycles, motorcycles and motorised vehicles. The transport fares charged depend on distance covered, type of vehicle (whether motorised vehicle, motorcycle or bicycle) and time of the day (usually higher fares are charged at night).

In urbanized settings, the transport providers are often organized in formal associations of owners and drivers under a national umbrella organization-the Uganda Taxi Operators and Drivers’ Association (UTODA). This association acts as a regulatory organ and plays a major role in the collection of income tax from this informal sector. They try to enforce discipline and hygiene through fines, suspension of membership and withdrawal of the right to operate, among other possible sanctions. For security and safety concerns, the association tries to ensure that members wear uniforms and/or helmets, although compliance has been mixed, mainly due to the high cost of meeting the set requirements. Members of the association pay an annual membership fee of between 6,000-10,000 Uganda shillings (Ug sh) ($2.72 - 4.50 United States Dollars (USD)), and also pay an operational licence (tax) [6]. In the rural areas, however, such formal associations are not common. Providers are organized into small informal associations with poorly defined roles and loosely-defined membership[6].

### Informal transport for maternal services in the context of health markets

**Figure 1:**
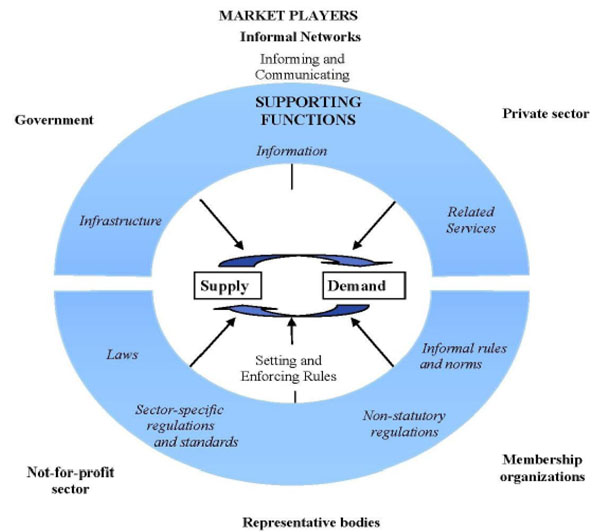
**Health service delivery in the context of a health market.** Source: Adapted from Elliott *et al.*, 2008, Figure 3.

A lot of activity in the health sector in developing economies occurs outside formal regulated frameworks, with different players operating at different points/levels in the delivery of health goods and services. Figure 1 provides a conceptual framework of these different actors in the health service delivery system, and how they interface with each other to provide the ultimate health good and service [9,10].

At the centre, is a set of exchanges between providers and consumers, operating under both formal and informal rules and with supporting functions that offer an operating environment for actors, including access to information, physical infrastructure and services including transport. Improving health service delivery requires focus not only on the formal structures, but also the challenges existing in the informal structures and the actors therein who provide the bulk of related services.

## Methodology

The pilot was conducted in Kamuli, one of the two districts of Eastern Uganda where the quasi experimental study is being implemented. The two districts were selected out of a total of four districts in the eastern region that had at least 3 health sub districts (HSD). They were selected because they were more comparable in terms of their capacity to offer maternal health services. Kamuli has an estimated population of 680,500, with 35.9% of the population below the poverty line and 34% of pregnant women delivering in health facilities. Pallisa, which is the second district, has an estimated population of 480,000, 35.9% of its population live below the poverty line and 44% of women delivering in health facilities. In each district one of the three health sub-districts was randomly selected as an intervention HSD and one of the remaining two, which most closely reflected demographic composition and availability of health services infrastructure (both public and private), selected as a control HSD. In Kamuli, Budiope HSD with 14 health facilities is the intervention and Buzaaya HSD with 11 health facilities is the control. A HSD is based on a health administrative area with population ranging from 30,000 to 100,000 and up to 10 health facilities.

### Description of the study components

The intervention package comprises of a transport voucher that facilitates women to access free transport for maternal health services (Antenatal care, delivery care and postnatal care) and a service voucher that also facilitates access to these maternal health services. This package is given to all pregnant women (irrespective of the trimester of the pregnancy), resident in the study area during the study period. The study also has a health systems strengthening component that is being conducted in both the intervention and control districts. This component comprises of training of health workers, supportive supervision and distribution of basic supplies and equipment in facilities in the intervention and control arms.

The study was piloted in Kamuli district between December 2009 and March, 2010. During this period voucher booklets were given out to over 12,000 mothers. Thereafter the distribution of vouchers was stopped. All the women who had received vouchers during the pilot period (December 2009 to March 2010) continued to receive the services that the voucher entitled them to; use of local transport to a health facility and access to services with no extra demands by providers (such as provision of gloves). Between April and May 2010 the pilot study results were reviewed and used to inform the design and implementation of a wider intervention planned to run until June 2011.

The voucher booklets were distributed at antenatal care (ANC) clinics. Each voucher booklet distributed during the pilot contained twelve **transport vouchers** for movement to and from the facility for antenatal, delivery and post natal care. It also contained seven **service vouchers** for antenatal, delivery and post natal care. The transport voucher entitled a pregnant woman to use locally available transport (motor cycle, bicycle) to the health facility for ANC, delivery and postnatal care services. However, the number of vouchers used per woman depended on her gestation period at enrollment (determines how many ANC sessions she can attend) as well as her choice of delivery place and whether or not she attended PNC. Women who were referred from a lower level facility to a higher level facility e.g., for a caesarean section or other complications, received a special voucher for transport that allowed them to use a motorized vehicle (public taxi or ambulance).

When a mother wanted to come to the clinic she had to contact any transport provider of choice either by phone or by word of mouth (within their reach). Sometimes the transporters made the initiative and went out to look for the mothers themselves. After transporting a client, the transporter received a transport voucher from the client. The transporter retained this voucher and redeemed it for cash from the study coordinator. Payments were made to the transporters every two to five weeks at the stages where they registered.

### Setting up of the scheme

Setting up the transport scheme was a long tedious process that required several visits to the field to identify the transporters, their registration, organizing them into groups, and selecting leadership to work with. There were 3 main stages, which included a) mapping of the transport providers, b) sensitization of providers about the transport scheme, and c) identification of transporters and signing of contracts.

A mapping of the study area was done to locate the different stages that existed, identify the chairmen for the stages and local prices charged to the nearest health facility. The prices ranged from 2,000 to 10,000 Ug sh (USD $0.90 to 4.50) for motor cycles, and from 500 to 1,000 Ug sh (USD $0.22 to 4.5) for bicycles.

Determining the rate at which the transporters should be paid was challenging to the project. The commercial fee that is charged usually fluctuates depending on the distance travelled, the time of day or night as well as the terrain of the place.

Recognizing that it would be difficult to determine precise distances travelled and to confirm the time of travel, the study team decided to set a flat price of 5,000 Ug Shs (approximately USD $2.27) for each trip to or from a health facility using a motor cycle or 2,000 Ug. Sh (USD $0.90) for each bicycle ride to or from a health facility.

After agreeing on the fares with the transporters, the study team met with the transporters and told them about the study purposes and its mode of operation. Those who were willing to participate were then registered, and their photographs and identification details were obtained so that they could be given identity cards. Each transporter signed a contract with the study team to operate under specified and agreed conditions, but also taking into account the laws governing transportation services in the country. They were also solely responsible for the safety of the clients whom they transported. Contracts were signed with a total of 592 transporters, of whom 211(35.6%) used bicycles, 379 (64%) motorcycles and 2 (0.33%) cars. All the transporters were young to middle aged males. There were no females, because cycle transport was seen to be an exclusively male occupation. The transport business was the main source of livelihood for the majority of the transporters, although earnings were generally low because of the low volume of clients.

### Data collection methods

The findings reported in this paper are from a combination of methods including a review of project documents, focus group discussions and analysis of facility level data. Four focus group discussions were conducted with transporters who consisted of motorcycle riders in Kamuli district. The two car owners who had been registered initially had stopped participating in the study, so they were not included. The majority of bicycle riders also dropped out because mothers preferred to use motorcycles; consequently it was difficult to get sufficient bicycle riders for a focus group discussion, so the discussions were eventually limited only to the motorcycle transporters. The focus group discussions comprised of 6-8 motorcycle transporters who were selected with the help of the stage chair person (Leader of the transporters in a specific location). Thematic analysis was applied to the focus group discussions, minutes of project meetings and reports. Facility level data was reviewed to obtain statistics on the utilization of antenatal, delivery care services. They have been presented as graphs and figures.

Ethical approval to conduct this study was provided by MakSPH research and ethics committee and the Uganda National Council for Science and Technology (UNCST).

## Results

In the following sections we describe the transport scheme, benefits and unintended consequences resulting from the scheme, as well as challenges encountered in the implementation of the scheme. The results reported cover the period from December 2009 when the pilot started to June 2010, when the implementation started. Although the vouchers were distributed for only 3 months (December 2009 to March 2010), women who received these vouchers continued to receive services even after March 2010. The pilot was done and results obtained in only one district (Kamuli).

### Benefits and unintended consequences arising from the transport voucher system

#### Increased utilization of maternal care services

**Figure 2:**
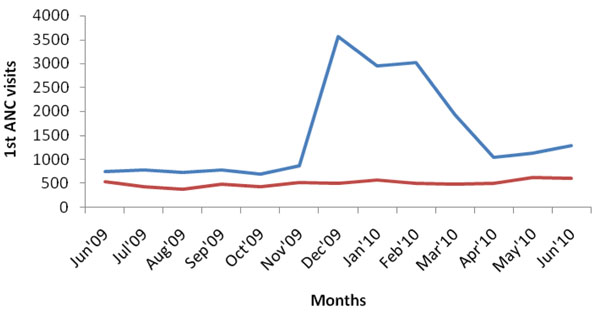
**1^st^ ANC Utilization in Kamuli District**.**** Blue line: intervention; red line: control

**Figure 3:**
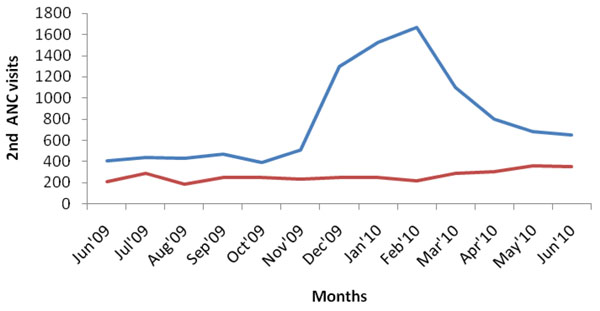
**2^nd^ ANC Utilization in Kamuli District.** Blue line: intervention; red line: control

**Figure 4:**
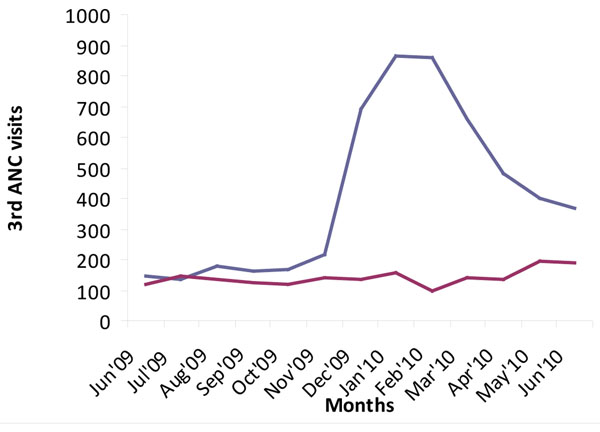
**3^rd^ ANC Utilization in Kamuli District.** Blue line: intervention; red line: control

**Figure 5:**
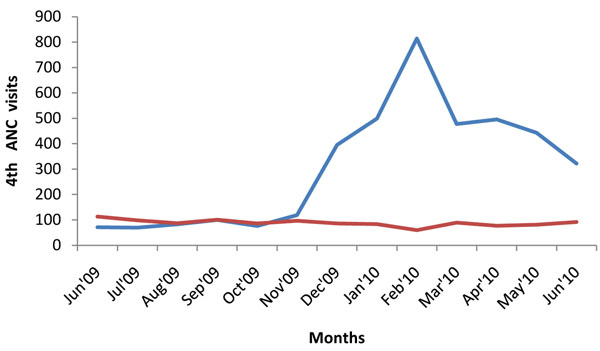
**4^th^ ANC Utilization in Kamuli District.** Blue line: intervention; red line: control

Although originally conceived by researchers from outside the district, the project was embraced by the community, and received active support from community leaders. The sensitization about the intervention increased community awareness about maternal health, with the transporters contracted by the project playing a leading role in mobilizing expectant mothers to attend services. This led to a dramatic increase in attendances at first, second, third and fourth visits for antenatal care (ANC), as well as an increase in institutional deliveries and postnatal care, and is described in more detail elsewhere [11]. Figures 2, 3, 4, 5 illustrate the increases in the utilization of maternal health services. This data was collected from facility utilization records from all the facilities that offer delivery care in the intervention and control areas from June 2009 to June 2010.

**Figure 6:**
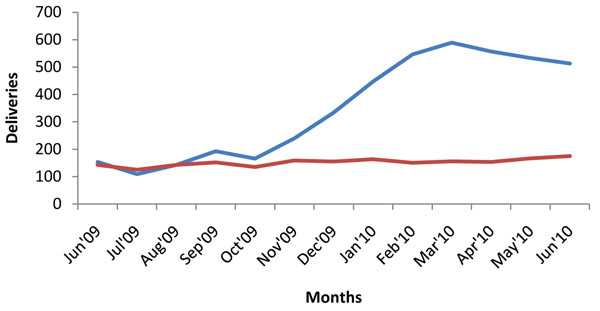
**Institutional Deliveries in Kamuli District.** Blue line: intervention; red line: control

What we observe is that the trend of service utilization in the control area is virtually the same overtime. However in the intervention areas, maternal service utilization (1st, 2nd, 3rd, 4th ANCs and delivery) increased sharply during the pilot period. ANC attendances declined when vouchers for transport were stopped (Figures 2, 3, 4, 5), but deliveries remained higher in intervention areas, probably due to the cohort of women who were given vouchers in the pilot phase (Figure 6).

#### Economic benefits

The project had economic spill-over effects to the transport providers, their families and community generally. The payment which the transporters received for the services they offered was the main source of income for the majority of them. It was especially high during the pilot phase of the project when the transporters were receiving 5,000 Ug sh (USD $2.27) per trip for each patient that they transported. However, the benefits received varied according to the level of participation of the provider and also according to the type of vehicle used by the provider as indicated in Tables 1 and 2. Transporters who had a very high level of participation as indicated by the number of mothers whom they transported (hence more vouchers) received an average of 437,538 Ug shs per month (USD $199). Those with low participation who transported few mothers (hence few vouchers) received only 12,200 Ug sh (USD $6). The bicycle riders (Table 2) also earned less than those who had motorcycles (Table 1).

**Table 1:** Payments to transporters using motorcycles in Kamuli district (December 2009 to June 2010)

Level of participation	Low	Moderate	Moderately high	Very high
Vouchers per individual	1 - 50	51 - 100	101 - 400	400>
Number of transport vouchers	1,597	1,614	17,461	31102
Number of transporters	83	21	77	43
Total paid in 7 months (Ug Shs)	7,088,000	6,737,500	73,226,000	131,699,000
Total paid in 7 months (USD)	3,222	3,063	33,285	59,863
Average amount per individual (Ug Sh)	85,398	320,833	950,987	3,062,767
Average amount per individual (USD)	39	146	432	1,392
				
Average amount paid per month per individual (Ug Sh)	12,200	45,833	135,855	437,538
				
Average amount paid per month per individual (USD)	6	21	62	199

*1 USD=2200 Ug.Shs

**Table 2:** Payments to transporters using bicycles in Kamuli district (December 2009 to June 2010)

Level of participation	Low	Moderate	Moderately high
Vouchers per individual (range)	1 - 50	51 - 100	101 - 400
Number of transport vouchers	293	195	256
Number of transporters	14	3	1
Total paid in 7 months ( Ug shs)	598,000	296,000	954,500
Total paid in 7 months (USD)	272	135	434
Average amount per individual (Ug sh)	42,714	98,667	954,500
Average amount per individual (USD)	19	45	434
			
Average amount paid per month per individual (Ug sh)	6,102	14,095	136,357
Average amount paid per month per individual (USD)	2.77	6.41	61.98

*1 USD=2200 Ug.Shs

Transporters generally reported that they were happy with the project and had benefitted financially from the scheme as illustrated by the selected quotes below from the Focus Group discussions conducted with transporters.

“……..this project helped us to sustain my family, I have managed to buy a bicycle from this project and other people have managed to buy motorcycles out of this project. Some people have opened up small loan schemes whereby we give ourselves loans amongst ourselves. So before this project came, our lives were not doing well but we are doing very well now……” [FGD Motorcycle riders Kamuli District]

“………for me my life has greatly changed as a result of this project. Before this project I was badly off but I have now managed to secure another motorcycle out of this project, I have bought 2 cows out of this project; I am also plastering my house and I am managing to pay fees for my children out of the money I am getting from this project and I have managed to sustain my family very well……….” [FGD Motorcycle riders Kamuli District]

In March 2010, the payment rates were reduced to between 2,000 and 5,000 Ug sh (USD $0.90 and USD $2.27) in an attempt to reduce the project costs. Consequently the earnings of the transporters decreased and some of them expressed disappointment with these revised rates as noted in the flowing expression below from the focus group discussion with transporters:

“………at first this project was good because the payment was good, but when they reduced it, we have also lost morale because of the small pay.” [FGD Motorcycle riders Kamuli District]

The costs incurred by the transporters included fuel, repairing their bicycles and motor cycles and paying their bosses (owners of the bicycles and motorcycles). The cost of fuel increased during the study from about Ug.sh 2,500 (USD $1.13) per litre to Ug.Sh 3,400 (USD $1.50) per litre, mainly due to the depreciation of the Uganda Shilling against the United States dollar against which fuel prices are denominated.

“…….in the past fuel was not very expensive like these days, but now fuel is very expensive but the charges are very low because we used to buy fuel at Ug.Shs 2,500 but it is now Ug.Shs 3,400. So we are not working on the project now.” (FGD Motorcycles Kamuli)

Lastly, one of the unintended consequences reported was that this resulted in crowding out of the other private transport service users, because the pregnant mothers were seen as better paying customers.

### Challenges to program implementation

#### Costs of running the voucher program

The costs presented here reflect the costs incurred from December 2009 to June 2010. Transport provider costs cover the cost for the payments made to the transporters. The administrative costs include the cost of per diem for the field coordinator who paid the transporters, as well as fuel and vehicle hire costs. In addition minor costs such as those for printing the vouchers, the identification cards and the contracts are also included. The overhead costs that were spent in processing the payment are not reflected as they were covered as part of the overall project administration which included many other elements and could not easily be attributed. The total cost of the project was USD $180,406 (See Table 3). Although desirable, a detailed cost analysis is beyond the scope of this paper.

**Table 3:** Costs of the transport voucher component (December 2009 – June 2010)

Item	Costs in Ug. Shs*	Costs in US$
Transporter provider costs	376,599,520	171,182
Administrative (covered as project costs elsewhere)	-	-
Printing Vouchers	8,691,750	3,951
Facilitation/supervision of Payment process	9,840,000	4,473
Printing identification cards	1,700,000	773
Stationery/photocopier for contracts	60,000	27
Grand Total	396,891,270	180,405

*1 USD=2200 Ug.Shs

#### Method of payment and verification of vouchers

The easiest method of reimbursement would have been to use bank accounts. However, the banks are located in the more urban areas of the district so they were not easily accessible to the majority of the transporters. Secondly, some of the transporters don’t own the transport vehicles that they use, so they used to hire them and make payments very frequently, in some cases even daily. Therefore they thought the use of a bank would create mistrust between them and their bosses. Having preferred cash payments, it was always challenging to process payments, given the administrative processes involved that often delayed the payments. The administrative processes involved auditing and verifying vouchers for claims to avoid duplication and forgery, visiting the field for each round of payment, among others.

#### Difficulty in obtaining operating licenses

Many of the transport providers did not have all the required legal documentation. They argued that it is a very expensive process and requires a lot of time. Secondly, because they usually operate deep in the villages where law enforcement officers are not within their reach, they are reluctant to process the licenses. However, when they have to transport mothers to urban centers, they get arrested. The study team subsequently undertook measures to ensure that all the transporters registered with the study obtain licenses. Below are some quotations from the focus group discussions with transporters.

“…….with the license we are not incurring any cost but it is our responsibility to pay for it because we operate in the village here they don’t ask for the license, but when you go to Kamuli they ask for it. One day they gave me a lady who was referred to Kamuli but as I was approaching Namalamba, they told me not to continue to Kamuli that the traffic officers were very serious arresting whoever could pass. So I had problems taking this woman up to Kamuli; I had to pay another person who had license to take this woman up to Kamuli”[ FGD Motorcycles, Kamuli District]

***“………*** *We are supposed to pay for the license but we don’t pay; they don’t arrest us, there are not many police traffic officers here unless you go to Kamuli. …… the moment you reach Buyende, they arrest you.* ***”*** *[FGD Motorcycles, Kamuli District]*

#### Changes in external conditions

Contracts with the transporters were signed for a predetermined period. However, changes in external conditions may occur and have an immediate effect that affects the suggested transport rates, making them appear unacceptably low. For instance there was a hike in the fuel prices that lasted over a month. Local changes such as increased rainfall during the Elnino rains also complicated the process further. Some transporters therefore stopped working as a result of this and the project had to continue negotiating with the providers. This was expressed in the quotations below.

*“………when the project came we said that let us transport these people because they are in problems as we also get some money but since they changed the prices of the transport we have also resorted to our old system of transporting whoever comes because you cannot work in losses because you are buying fuel Ug. Shs 3,400 a litre you can’t transport someone at Ug. Sh 3,000 yet some of us we are not the owners of of the motorcycles; we have our bosses who need their money at the end of the week.”* (FGD Motorcycles Kamuli)

#### Delays at the health facilities

The transport providers also complained about the delays that they encountered at the health facilities. They would have preferred to take the mother to the unit, wait for them and then return her back home. However, as a result of the high turn up of mothers and the shortage of health workers, at times the transporters had to wait for long periods for the mothers to be attended to.

## Discussion

The project made transport services more accessible to the majority of women in the community with an increase in the number of women attending ANC services for all the four visits. Most mothers tended to use the motor cycles rather than the bicycles. This was probably because the motor cycle boda-boda were more comfortable and they could move longer distances than the bicycles. Indeed previous studies have shown that motorcycles are preferred even though they tend to be more expensive. This increased availability and use of transport services could be attributed to two factors; a) the reduced cost of transport (the mothers didn’t have to pay any money), and b) easier accessibility to the transporters. Because of the financial incentives, the transporters were very active in mobilizing mothers to attend services through either their cell phones or physically reaching prospective clients.

The increased access to services that was noted in this study shows that lack of transport was a major contributing factor to non use of health facilities by women especially the poor. According to previous research the poorest segments of the population tended not to use motorcycle boda-boda because they could not afford it [6]. Initiatives that can help to reduce the cost of transport could therefore go a long way in increasing access to health services by the poor. However, this requires government interventions such as reducing license fees and other forms of levies and streamlining the registration processes, to allow more providers to obtain the required license. This would allow more entrants into the field, with the creation of more competition, and perhaps a lowering of the transport charges and eventually more affordable services.

Economic gains achieved by those engaged with the project have been recorded. Given that most of the transporters are generally school drop outs that have difficulty finding regular employment, the project acted as an opportunity to boost their income. Similar studies have shown that income earned through these boda-boda services provide benefits not only to the transport provider and his immediate family but also to dependants [6,7,10]. Other beneficiaries include groups that provide complementary services such as those who repair motorcycles, or who provide fuel. Developments in this sector can therefore provide economic gains beyond the increased income for the transport provider.

This kind of transport has, however, been associated with some risks. These include thefts of motorcycles at night, at times even accompanied by loss of life, accidents stemming from reckless or drunk riding or simply poor riding skills [6]. However, in this study accidents did not seem to be a major problem, partly perhaps because the transport providers had signed a contract which indicated that irresponsible behavior could lead to termination of the contract. There are laws that require that riders have licenses, wear helmets and go to a training school where they can be taught how to ride. However, these regulations have often not been enforced [6]. The associations for the providers could provide some help in ensuring that these laws are enforced. However, it has been reported that these associations are often informal and so they tend to play more of a voluntary role and therefore find it difficult to enforce such laws. According to Bloom et al [10], compliance with rules may be affected by the legitimacy of the body trying to enforce the rules. The establishment of franchises in this sector could also help to promote ethical behavior in terms of compliance with the transport laws and regulations[10]. Bishai and colleagues found that franchising provides more incentives for local entrepreneurship [12]. A franchise could encourage provision of safe efficient transport services. This could promote the development of trust in reputable providers, leading to an increase in their client base. Hence eventually such arrangements could benefit both the providers and the clients whose safety would be more assured. Regarding the insecurity associated with night travel, the local governing authorities could play a more significant role in ensuring that riders and mothers are safe at night through enhancing local security that can curtail the activities of robbers.

In considering whether this initiative can be scaled up, two important issues warrant discussion; a) the capacity to run the scheme, and b) the cost of running the scheme. Proper running of the scheme requires that there is a clear system for paying the providers. This payment needs to be done regularly and frequently because these transporters handle small sums of money and therefore need the money for running costs. This problem is likely to be magnified in the early stages of the scheme when there is a lot of suspicion. After trust has been gained then payments can be made monthly. To reduce transaction costs, the use of mobile phones for making payments could be explored. However, such systems are usually available in the more urban areas and almost nonexistent in the rural, primarily because there is no business in the rural areas. Lastly, a system for identifying fraud also needs to be set up. Fraud is a common problem with voucher programs. The voucher agency has to ensure that the vouchers have special security features that cannot be forged easily. A mechanism for confirming that services have been supplied will also be necessary before the transport providers are paid.

The second main issue is the cost of the project and therefore concerns of sustainability. In a resource poor country, the government may have difficulties meeting the financial requirements, and donors get fatigued over time. It is therefore necessary to think of ways of mobilizing the community to contribute to the running of the programme. One option could include the creation of a community mobilization fund perhaps through community insurance schemes. Families could then be encouraged to make periodic contributions that would cater for the transport needs of the mother and the newborn. This insurance scheme would then liaise with local transport providers and discuss pricing and service arrangements.

Ownership of the project could also affect its sustainability. It was conceived by a research team from outside the district. However, the study was implemented in close collaboration with the district health team. Regular meetings were held with stakeholders at district level to discuss the implementation of the study and to address the challenges that arose, including soliciting for ways of sustaining the project beyond the study phase.

In conclusion, the project has shown that it is possible to utilize locally available transport systems that are responsive to the different local and rural contexts. Such low cost intermediate transport systems (bicycles and motorcycles) can reduce the challenges paused by geographical inaccessibility and poor transport networks. Secondly, they are effective in increasing institutional attendances for maternal services and hence can lead to improvements in maternal and child health. An effort should be made to identify ways of reducing the cost of transport by encouraging more investment in the sector, with the hope of increasing competition and hence reduction of user charges. However, care must be taken to ensure that there is enforcement of laws that will ensure the safety of passengers and the transport providers themselves.

## List of abbreviations used

TBA: traditional birth attendant; MakSPH: Makerere University College of Health Sciences; FHS-RPC: Future Health Systems Research Program Consortium; MU-JHU: Makerere University - Johns Hopkins University; UTODA: Uganda Taxi Operators and Drivers’ Association; USD: United States Dollars; HSD: health sub-district; ANC: antenatal care; UNCST: Uganda National Council for Science and Technology; FHS: Future Health Systems; DFID	U.K.: Department of International Development.

## Competing interests

The Authors declare no competing interests.

## Authors' contributions

EEK, CM and GWP contributed to the formulation of the study and co wrote drafts of the manuscript. GB and HL also contributed to the formulation of the study, and provided substantial inputs into the manuscript. DHP, DS, FS, OO and RH reviewed and provided substantial inputs into the manuscript. All the Authors read, provided substantial input and approved the final manuscript. EEK and CM are guarantors of this paper.
